# Breaking the Taboo: Illicit Drug Use among Adolescents with Type 1 Diabetes Mellitus

**DOI:** 10.1155/2016/4153278

**Published:** 2015-12-29

**Authors:** Anna M. Hogendorf, Wojciech Fendler, Janusz Sieroslawski, Katarzyna Bobeff, Krzysztof Wegrewicz, Kamila I. Malewska, Maciej W. Przudzik, Malgorzata Szmigiero-Kawko, Beata Sztangierska, Malgorzata Mysliwiec, Agnieszka Szadkowska, Wojciech Mlynarski

**Affiliations:** ^1^Department of Pediatrics, Oncology, Hematology and Diabetology, Medical University of Lodz, 91-738 Lodz, Poland; ^2^Department of Studies on Alcoholism and Other Dependencies, Institute of Psychiatry and Neurology, 02-957 Warsaw, Poland; ^3^Students' Scientific Circle at the Department of Pediatrics, Oncology, Hematology and Diabetology, Medical University of Lodz, 91-738 Lodz, Poland; ^4^Department of Pediatrics, Diabetology and Endocrinology, Medical University of Gdańsk, 80-211 Gdańsk, Poland

## Abstract

*Background*. The aim of the study was to explore the prevalence of illicit drug use in a group of Polish adolescents with type 1 diabetes (DM1) in comparison with a national cohort of their healthy peers.* Methods*. Two hundred and nine adolescents with DM1, aged 15–18 years, were studied in 2013 with an anonymous questionnaire prepared for the European School Survey Project on Alcohol and Other Drugs (ESPAD). The control group was a representative sample of 12114 students at the same age who took part in ESPAD in 2011. Metabolic control was regarded as good if self-reported HbA1c was <8% or poor if HbA1c was ≥8%.* Results*. Lifetime prevalence of illicit drug use was lower among adolescents with DM1 than in the control group [58 (28%) versus 5524 (46%), *p* = 10^−5^]. Cannabis preparations were the most frequently used substances [38 (18.3%) versus 3976 (33.1%), *p* = 10^−5^], followed by tranquilizers, sedatives, and amphetamine. Lifetime and last 12-month use of cannabis were associated with poorer glycemic control (HbA1c ≥ 8%), *p* < 0.01 and 0.02, respectively.* Conclusions*. Adolescents with DM1 report using illicit drugs to a lesser extent than their healthy peers. The use of cannabis is associated with a poorer metabolic control in teens with DM1.

## 1. Introduction

Experimental behaviors are a characteristic feature of adolescence. Growing evidence suggests that adolescents with chronic conditions, including type 1 diabetes mellitus (DM1), are likely to engage in risky behavior to at least similar, if not greater, extent than their healthy peers [1, 2]. However, drug abuse or even single experimental use of recreational drugs may be especially dangerous in patients with DM1, due to inability to self-manage diabetes [3]. This may contribute to increased morbidity, mortality, and healthcare costs associated with acute diabetes-related events [4–8].

Despite the medical and social importance of the problem, it seems that the topic remains a taboo in families and is underrecognized or easily neglected in complex medical management [9]. Current medical literature contains little data on the prevalence of drug use and abuse in type 1 diabetes, as only a few case reports and a small number of methodologically varying and incomparable analyses are available [2, 3, 10–14]. The problems with conducting such surveys are collecting a proper sample size and an appropriate reference group recruited from the community, over- or underreporting, and the use of self-report questionnaires that are less reliable in clinically recruited samples.

We aimed to evaluate the prevalence of illicit drug use among Polish adolescents with DM1 and to compare it with the habits of healthy peers from a large national cohort, participating in the European School Survey Project on Alcohol and Other Drugs (ESPAD).

## 2. Patients and Methods

### 2.1. DM1 Group

Adolescents with DM1 were studied in May and June 2013 in three diabetes centers: Department of Pediatrics, Oncology, Hematology, and Diabetology, Medical University of Lodz, Department of Pediatrics, Diabetology, and Endocrinology, Medical University of Gdańsk, and Diabetes Outpatient Clinics in Sanok area. The study comprised patients scheduled for a routine visit in each of the above sites during the study period (May-June 2013), born between 1994–1997, and with at least one-year history of diabetes. To ensure complete anonymity, the patients were recruited by medical students, not involved in diabetes management. The subjects and their parents had been informed about the aim of the study, its anonymous, and voluntary character and were allowed to ask questions. Written informed consent was obtained before the inclusion in the study. Confidentiality and anonymity were warranted by asking the patients to fill in the questionnaires in separate rooms, without the presence and supervision of their parents or diabetic team members. After completing the questionnaires, the patients were asked to deposit closed envelops with their response sheets into a box which remained closed until the end of the study.

The questionnaire contained initial questions regarding the course of diabetes, and the main standardized questionnaire used in the Polish edition of ESPAD, conducted in May and June 2011. The ESPAD is a collaborative effort of independent research teams in more than forty European countries and the largest cross-national research project on adolescent substance use in the world. The program was launched in 1995 and the surveys are repeated every four years. The aim of ESPAD is to collect comparable data on substance use among 15-16- (and in some countries also 17-18-) year-old students in as many European countries as possible. Poland has been collecting ESPAD data since 1995. The methodology of the survey, including the questionnaire, is described in detail elsewhere [15]. Briefly, the surveys are conducted with common group-administered questionnaires. The students answer the questionnaires anonymously in the classroom with teachers or research assistants functioning as survey leaders. The 2011 Polish sample of classes was nationally representative. To avoid seasonal variability, data was collected in spring (in May and June). Participants were divided into two subgroups, depending on their age (15-16- and 17-18-year-olds, resp.).

We retrieved and analyzed only these questions from the ESPAD questionnaire which regarded lifetime use of illicit drugs, such as cannabis (marijuana and hashish), ecstasy, amphetamines, cocaine, crack, LSD or other hallucinogens, heroin, gamma hydroxybutyrate (GHB), tranquillizers or sedatives without a doctor's prescription, inhalants, magic mushrooms, anabolic steroids, and Polish heroine (a crude preparation of heroin made from poppy straw intended for injection). Because some adolescents tend to pretend to have used drugs, the nonexistent dummy drug “*Relevin*” was included among real drugs in the questionnaire in order to test the validity of the survey.

Metabolic control was assessed by asking the patients to indicate the interval (6–8%, 8–10%, 10–12%, and >12%) in which the mean value of their last three HbA1c measurements was found. It was regarded as good if HbA1c <8% or as poor if ≥8%.

### 2.2. Control Group

The control group was a representative sample of 12144 Polish students, aged 15–18 years, born in 1992–1995, who participated in the fifth data collection of ESPAD in May and June 2011. The survey was performed as a written questionnaire during school time, according to the ESPAD Protocol [15].

Our study was approved by the Bioethics Committee of the Medical University of Lodz.

### 2.3. Statistical Analysis

Differences in the prevalence of illicit drug use were evaluated using Pearson's Chi-square test. Odds ratios with a 95% Confidence Intervals were also calculated where appropriate. Differences between DM1 and control groups for continuous variables were assessed using Mann-Whitney *U* test. Comparisons with *p* values lower than 0.05 were considered as statistically significant.

## 3. Results

### 3.1. Participants

In the three participating centers there were 400 patients treated for type 1 diabetes, aged 15–18 years. However, out of these 400 adolescents, 175 were not scheduled for a visit in the clinic between May and June 2013 and could not be included in the study. 16 of remaining 225 eligible patients refused to participate, which was reportedly motivated by the lack of time to complete the questionnaire. The acceptance of participating amounted to 92.9% and so 209 patients returned the questionnaires. Characteristics of teenagers with DM1 and the control group are shown in Table 1.

The DM1 and the control groups had similar gender and age distribution. The mean ages of the DM1 and the control groups members were 16.5 ± 1.0 and 16.9 ± 0.9 years, respectively (*p* = 0.4).

The mean duration of diabetes was 6.5 years ± 4.4. Half of the DM1 patients (53%) had HbA1c level above 8%.

Lifetime prevalence of illicit drug use was significantly lower among adolescents with DM1 than in the control ESPAD group: 58 (28%) versus 5524 (46%); *p* < 10^−5^; odd ratio OR (95% CI) = 0.46 (0.34–0.62). This held true for all drugs in the ESPAD survey (Table 2). Moreover, some adolescents tried several illicit substances over the course of their adolescent years. Cannabis was the most commonly used illicit drugs among adolescents in both groups: 38 (18.3%) versus 3976 (33.1%), *p* = 10^−5^. A much smaller percentage reported using amphetamine: 8 (3.9%), LSD and other hallucinogens were mentioned by 3 (1.4%), cocaine was mentioned by 3 (1.4%), and magic mushrooms was mentioned by 3 (1.4%) of the DM1 patients, and the rates for ecstasy 1 (0.5%), crack 1 (0.5%), heroin 1 (0.5%), and gamma hydroxybutyrate (GHB) 1 (0.5%) were even lower. Interestingly, tranquilizers and sedatives without medical supervision were used by 20 (9.6%) of teens with DM1 versus 1911 (15.9%) of controls (*p* = 0.01), more frequently by girls than boys. The use of nonexisting “*Relevin*” was reported by 0 (0%) of the patients versus 157 (1.3%) of the students, *p* = 0.17.

Sex differences were evident in the DM1 group. Male adolescents, as shown, were more likely than their female counterparts to use illicit drugs (30.5% versus 25.7%). Girls with DM1 reported the use of cannabis, amphetamine, and tranquillizers only.

The median age at first consumption of cannabis, tranquillizers, amphetamine, ecstasy, and inhalants was similar in both groups (Table 3). Similarly as in the general population group, inhalants were the first tried psychoactive substances used in the DM1 group.

There were no statistical differences between the level of HbA1c in patients who admitted or denied lifetime experimenting/using any of the drugs, *p* = 0.1438.

**Figure 1:**
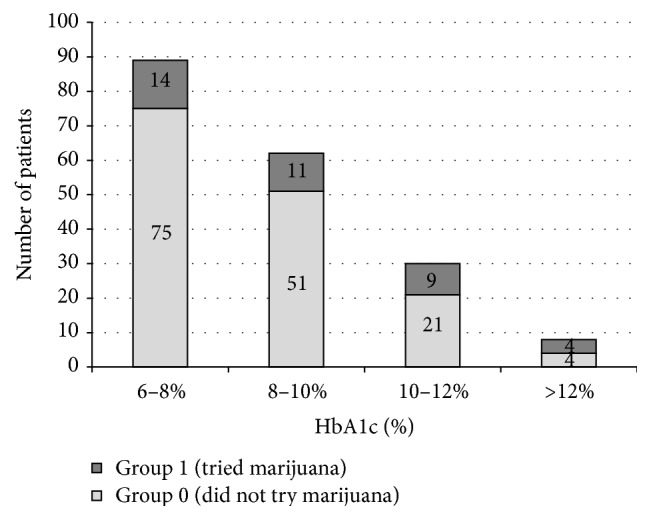
The proportion of patients who tried or did not try marijuana, according to HbA1c levels, *p* = 0.03; response rate to that question was 189/209.

However, lifetime and last 12-month use of marijuana were associated with poorer glycemic control (HbA1c ≥ 8%), *p* < 0.01 and 0.02, respectively. The proportion of patients who tried or did not try marijuana, according to HbA1c levels, is shown in Figure 1.

No significant associations were found for duration of diabetes and use of any drugs or marijuana in particular, even after adjusting for patients' age and sex (*p* = 0.22).

## 4. Discussion

Illicit drugs have acute detrimental effects that are often fatal in healthy young people [16]. In patients with type 1 diabetes, their use may thoroughly disrupt diabetes management and precipitate acute and chronic complications. Stimulants are also likely to cause or mask many mental disorders that are more often encountered in DM1 patients than in general population [16–19].

Some of recreational drugs have a direct influence on glucose metabolism. Amphetamine, ecstasy, or cocaine increases the release of catecholamines, cortisol, and other contraregulatory hormones that enhance gluconeogenesis, glycogenolysis, and lipolysis and are associated with reported episodes of diabetic ketoacidosis (DKA) [20–22]. Cocaine and heroin abuse has been reported to cause hyperglycaemic hyperosmolar state [23] and to be the strongest independent risk factor for recurrent DKA [21, 22, 24]. Androgenic-anabolic steroids (AS), taken orally or by injection at doses much higher than would be prescribed, increase the risk of early heart attacks, strokes, liver tumors, kidney failure, serious psychiatric problems, and long-term effects [25]. Regular use of GHB may lead to Cushing's syndrome [26, 27]. The health-related harms of cannabinoids use differ from those of other drugs in that they contribute little to mortality. However, cannabinoids impair judgment and cause food cravings or loss of appetite which are likely to have a negative effect on self-management behaviors (e.g., carbohydrate counting). Chronic use of cannabis may reduce motivation to maintain good metabolic control [28] and may increase the risk of neurologic or psychiatric disorders [29, 30].

In our study, the prevalence of illicit drug use was only half as high among adolescents with diabetes than in the healthy controls. This proportion held true for both age groups: 15-16- and 17-18-year-olds, which may indicate a better health awareness in the group of DM1 patients and/or a better parental control.

Teenagers with DM1 confessed using a wide range of illicit drugs, including those taken intravenously. Like in the general population and as shown in other studies, the most popular was marijuana. Male adolescents were more likely to use illicit drugs compared to their female counterparts. Girls with DM1 reported the use of only cannabis, amphetamine, and tranquilizers or sedatives. None of the DM1 girls admitted experimenting with “hard” drugs. However, it is notable that more girls than boys with DM1 reported the use of tranquillizers or sedatives for nonmedicinal purposes but still fewer than the healthy controls. Tranquilizers or sedatives are a widely used group of prescription medication; however, these drugs may also be used for the purpose of “getting high” rather than for medical reasons. In the ESPAD survey nearly half of the examined students in Poland (48%) admitted that both tranquilizers and sedatives were easily available.

Our study had several strong sides, including the use of a validated questionnaire, proven in the ESPAD surveys since 1995, and a large national control group of 12114 healthy students. The investigated substance use habits of Polish students turned out to be similar to those of the European average in students who participated in the ESPAD survey in 2011. One may argue, however, that, due to the unwillingness of adolescent patients to confess a risky behavior, self-reported data might underestimate the problem and limit the validity of the survey. We found it crucial to diminish the risk of underreporting by giving the patients a feeling of complete anonymity. Therefore, the questionnaires were collected by medical students not involved in the diabetes patients management. Owing to that, the participation and response rates were very high, as only 16 out of 225 patients refused to take part in our study. When it comes to validity measures, the use of the nonexistent dummy drug was reported by none of the patients, making the survey reliable.

The study, however, did have some limitations. The first was a relatively small sample size in comparison with the large control group, which may have influenced its statistical power. To avoid bias caused by different patterns of substance use by DM1 adolescent patients throughout the school year, we were able to enroll only the patients scheduled for the outpatient clinical visit in May and June (209 out of 400), according to the Polish ESPAD Protocol. However, in spite of the strict inclusion criteria, the study group contained over 50% of DM1 teens in the three study sites (from around 2000 pediatric patients), that is, 12–14% of all Polish pediatric patients with type 1 diabetes.

The second constraint was the metabolic control, performed only with the patient-reported mean value of the last three HbA1c measurements and no DKA-related questions were added. This, however, gave the participants an enhanced sense of anonymity. Moreover, due to our observations that adolescent people seldom remembered their last HbA1c, the patients were asked to indicate the interval (6–8%, 8–10%, 10–12%, and >12%) in which the mean value of their last three HbA1c measurements was found. Therefore, the metabolic control was regarded as good if HbA1c <8% or as poor if ≥8%, a value close to the limit of good metabolic recommended by ISPAD and ADA (HbA1c < 7.5%). Nevertheless, possibly due to the lack of exact HbA1c values, we were able to show the association of worse glycaemic control with lifetime and 30-day use of marihuana only. Other authors observed clearer association between overall drug use, worse glycaemic control, and a higher risk of diabetic ketoacidosis [3, 20].

Our results are more encouraging than the ones obtained in other countries (Table 4). In an Italian study, the overall drug use was shown to be slightly higher in T1D group. Female adolescents with DM1 exhibited even a higher rate of consumption of all illicit drugs studied than the healthy peers, while in male patients the rate was similar to the controls [2]. A survey conducted by Martínez-Aguayo et al. showed that lifetime illicit drug use by older DM1 students (in the 11th through 12th grades) approached the Chile national average. Lower rates (9.6% versus 22%) were observed only in younger students (in 8th through 10th grades) [12]. In a British postal questionnaire study, 29% of young diabetic patients (16–30 years of age) reported using street drugs, and 68% of them used them more than once a month [13].

Lifetime prevalence of illicit drugs among young Australians with DM1 was 77%, and 47% of them admitted using them within the last year. Recreational drug use was the most common among persons under 20 years (80%). Among those who used drugs, 24% reported daily use and 68% were polydrug users [3].

The observed inconsistency of results from various studies on illicit drug use among adolescents with type 1 diabetes mellitus is mostly due to methodological differences as well as different time of performing them. It is difficult to compare the results from the present study with those obtained 10–30 years ago [10, 11]. Variations may also result from the overall discrepancy of the prevalence of drug use in different countries. For example, according to ESPAD, countries like Czech Republic, France, and Monaco have the highest prevalence in Europe while in many Balkan countries and Norway the problem is less frequent [31].

Although the initiation time of drug use, as shown in our study, was similar in the clinical and control groups, the data indicate that better preventive strategies should be introduced as early as possible (even in children under 10 as the first use of inhalants starts at 10.5 years). The high rate of unawareness (up to 72%) of the adverse effects of illicit drugs on diabetes among young patients with DM has been reported in literature [13]. Therefore, proper education and the early introduction of prevention programs are necessary. Adolescents with diabetes should be regularly encouraged to refrain from drugs and be given this information through a friendly dialog at each visit. Because only a small number of patients inform health professionals about drug use [13], doctors should be able to recognize signs of recreational use or addiction and organize regular screening, especially in those with poor glycemic control and those who experience recurrent ketoacidosis.

## 5. Conclusions

This study showed that adolescents with T1D use recreational drugs less frequently than their healthy peers. The use of cannabis is associated with a poorer metabolic control in teens with DM1. Illicit drug use prevention must be an integral part of medical care for teenagers with DM1 and intervention introduced as early as possible.

## Figures and Tables

**Table 1:** Clinical characteristics of patients with diabetes and controls.

	Diabetic patients (*n* = 209)	Healthy controls (*n* = 12144)	*p* level
Female (%) versus male (%)	102 (48.8) versus 107 (51.2)	5982 (50.6) versus 6132 (49.3)	*p* = 0.6

Age (year) Mean ± SD	16.5 ± 1.0	16.9 ± 0.9	*p* = 0.4

15-16 years (%)	98 (47.1)	6050 (49.9)	

17-18 years (%)	110 (52.8)	5055 (50.0)	

Diabetes duration (year) Median (interquartile range)	6 (3–10)	—	

HbA1c (%)^*∗*^		—	
6–8%	89 (47)		
8–10%	62 (33)		
10–12%	30 (16)		
>12%	8 (4)		

Insulin (units/day) Median (interquartile range)	50 (30–65)	—	

Insulin pump therapy (%)^*∗∗*^	79/183 (43)	—	

^*∗*^Response rate of 189/209; ^**∗∗**^response rate of 183/209.

**Table 2:** Lifetime prevalence of illicit drugs use in 209 teenage patients with T1D compared with their healthy peers (*n* = 12114).

Stimulant	Subjects	Lifetime prevalence		*p*
		DM1 *n* (%)	C *n* (%)	
Illicit drugs	All	58 (28.2)	5524 (46.1)	**p** < 10^−5^
	15-16 years	22 (23.0)	2436 (40.7)	**p** = 0.0004
	17-18 years	36 (33.0)	3085 (51.4)	**p** = 0.0001
	Boys	32 (30.5)	2893 (49.0)	**p** = 0.0002
	Girls	26 (25.7)	2631 (43.3)	**p** = 0.0004

Marijuana/hashish	All	38 (18.4)	3976 (33.1)	**p** = 10^−5^
	15-16 years	12 (12.4)	1587 (26.5)	**p** = 0.0018
	17-18 years	26 (23.9)	2387 (39.8)	**p** = 0.0007
	Boys	24 (22.6)	2396 (40.5)	**p** = 0.0002
	Girls	14 (13.9)	1580 (26)	**p** = 0.006

Amphetamine	All	8 (3.9)	814 (6.8)	*p* = 0.127
	15-16 years	3 (3.1)	295 (4.9)	*p* = 0.546
	17-18 years	5 (4.6)	518 (8.6)	*p* = 0.188
	Boys	7 (6.5)	484 (8.2)	*p* = 0.669
	Girls	1 (1.0)	330 (5.4)	*p* = 0.083

LSD and hallucinogens	All	3 (1.4)	473 (3.9)	*p* = 0.097
	15-16 years	1 (1.0)	212 (3.5)	*p* = 0.287
	17-18 years	2 (1.8)	260 (4.3)	*p* = 0.301
	Boys	3 (2.8)	289 (4.9)	*p* = 0.446
	Girls	0 (0.0)	184 (3.0)	*p* = 0.140

Ecstasy	All	1 (0.5)	486 (4.0)	**p** = 0.015
	15-16 years	0 (0.0)	209 (3.5)	*p* = 0.110
	17-18 years	1 (0.9)	276 (4.6)	*p* = 0.111
	Boys	1 (0.9)	305 (5.1)	*p* = 0.081
	Girls	0 (0.0)	181 (3.0)	*p* = 0.145

Magic mushrooms	All	3 (1.4)	428 (3.6)	*p* = 0.147
	15-16 years	0 (0.0)	185 (3.1)	*p* = 0.142
	17-18 years	3 (2.8)	242 (4.0)	*p* = 0.671
	Boys	3 (2.8)	301 (5.1)	*p* = 0.399
	Girls	0 (0.0)	127 (2.1)	*p* = 0.267

Tranquillizers and sedatives	All	20 (9.6)	1911 (15.9)	*p* = 0.014
	15-16 years	8 (8.2)	906 (15.1)	*p* = 0.079
	17-18 years	12 (11.0)	1004 (16.7)	*p* = 0.114
	Boys	7 (6.5)	632 (10.6)	*p* = 0.227
	Girls	13 (12.9)	1279 (21.0)	*p* = 0.062

Crack	All	1 (0.5)	237 (2.0)	*p* = 0.199
	15-16 years	1 (0.9)	118 (2.0)	*p* = 0.763
	17-18 years	0 (0.0)	118 (2.3)	*p* = 0.263
	Boys	1 (0.9)	161 (2.7)	*p* = 0.414
	Girls	0 (0.0)	76 (1.3)	*p* = 0.500

Cocaine	All	3 (1.4)	439 (3.7)	*p* = 0.133
	15-16 years	2 (2.0)	196 (3.3)	*p* = 0.698
	17-18 years	1 (0.9)	242 (4.0)	*p* = 0.161
	Boys	3 (1.4)	247 (4.2)	*p* = 0.649
	Girls	0 (0.0)	192 (3.1)	*p* = 0.128

Heroine	All	1 (0.5)	275 (2.3)	*p* = 0.133
	15-16 years	1 (0.9)	150 (2.5)	*p* = 0.545
	17-18 years	0 (0.0)	124 (2.1)	*p* = 0.241
	Boys	1 (0.9)	166 (2.8)	*p* = 0.385
	Girls	0 (0.0)	109 (1.8)	*p* = 0.330

Drugs by injection with needle	All	1 (0.5)	212 (1.8)	*p* = 0.257
	15-16 years	1 (0.9)	98 (1.6)	*p* = 0.841
	17-18 years	0 (0.0)	113 (1.9)	*p* = 0.321
	Boys	1 (0.9)	142 (2.4)	*p* = 0.507
	Girls	0 (0.0)	70 (1.2)	*p* = 0.543

GHB	All	1 (0.5)	154 (1.3)	*p* = 0.481
	15-16 years	1 (0.9)	79 (1.3)	*p* = 0.837
	17-18 years	0 (0.0)	74 (1.2)	*p* = 0.469
	Boys	1 (0.9)	142 (2.4)	*p* = 0.769
	Girls	0 (0.0)	70 (1.2)	*p* = 0.759

Anabolic steroids	All	2 (1.0)	328 (2.7)	*p* = 0.179
	15-16 years	0 (0.0)	143 (2.4)	*p* = 0.226
	17-18 years	2 (1.8)	184 (3.1)	*p* = 0.271
	Boys	2 (1.8)	271 (4.6)	*p* = 0.271
	Girls	0 (0.0)	57 (0.9)	*p* = 0.651

Relevin	All	0 (0.0)	157 (1.3)	*p* = 0.178
	15-16 years	0 (0.0)	79 (1.3)	*p* = 0.489
	17-18 years	0 (0.0)	77 (1.3)	*p* = 0.449
	Boys	0 (0.0)	111 (1.9)	*p* = 0.287
	Girls	0 (0.0)	46 (0.8)	*p* = 0.770

**Table 3:** Initiation time of illicit drugs use in years of age.

Substance	DM1 (*n*)	Mean	Median	*Q*25–75%	Control (*n*)	Mean	Median	*Q*25–75%	*p* level
Marijuana/hashish	33	15.21	15.00	15.00–16.00	4084	15.32	15.00	15.00–16.00	0.6657
Tranquilizers	18	14.22	14.00	14.00–15.00	1894	14.58	15.00	14.00–16.00	0.1879
Amphetamine	6	15.50	15.00	14.00–17.00	864	15.16	16.00	14.00–17.00	0.9993
Ecstasy	1	14.00	14.00	14.00–14.00	498	14.76	15.00	14.00–16.00	1.0000
Inhalants	4	12.50	13.00	10.50–14.50	687	13.59	14.00	12.00–15.00	0.3810

**Table 4:** Prevalence of illicit drug use in young people with type 1 diabetes.

Authors [reference]	Year of publication	Country	Subjects (age)	Prevalence of drug use (%)	Methodology
Gold and Gladstein [11]	1993	USA	79 (11-12)	9%	Anonymous self-administered questionnaire, summer camps

Glasgow et al. [10]	1991	USA	101 (12–20)	25%	Anonymous self-administered questionnaire with verification by urine drug screening

Frey et al. [14]	1997	USA	155 (10–20)	10%	A descriptive cross-sectional design, self-report on routine clinic visit

Martínez-Aguayo et al. [12]	2007	Chile	193 (13–20)	10%	Anonymous self-administered questionnaire, diabetes summer camps

Ng et al. [13]	2004	UK	158 (16–30)	29%	Anonymous self-reported postal questionnaire

Lee et al. [3]	2012	Australia	506 (13–44)	77%	Radio broadcast/hospital advertising

Scaramuzza et al. [2]	2010	Italy	215 (12–16)	39,5% cannabis, 3,25% other drugs	Anonymous self-administered questionnaire, diabetes camps
